# Differentiation of Clear Cell and Non-clear-cell Renal Cell Carcinoma through CT-based Radiomics Models and Nomogram

**DOI:** 10.2174/1573405619666221121164235

**Published:** 2023-05-29

**Authors:** Delu Cheng, Yeerxiati Abudikeranmu, Batuer Tuerdi

**Affiliations:** 1 Department of Radiology, People's Hospital of Xinjiang Uygur Autonomous Region, Urumqi 83000, China;; 2 Department of Radiology, Liaocheng Traditional Chinese Medicine Hospital, Liaocheng, Shandong 252000, China

**Keywords:** Radiomics, computed tomography, machine learning, nomogram, clinicians, ccRCC

## Abstract

**Purpose:**

The aim of the study was to investigate the feasibility of discriminating between clear-cell renal cell carcinoma (ccRCC) and non-clear-cell renal cell carcinoma (non-ccRCC) *via* radiomics models and nomogram.

**Methods:**

The retrospective study included 147 patients (ccRCC=100, non-ccRCC=47) who underwent enhanced CT before surgery. CT images of the corticomedullary phase (CMP) were collected and features from the images were extracted. The data were randomly grouped into training and validation sets according to 7:3, and then the training set was normalized to extract the normalization rule for the training set, and then the rule was applied to the validation set. First, the T-test, T'-test or Wilcoxon rank-sum test were executed in the training set data to keep the statistically different parameters, and then the optimal features were picked based on the least absolute shrinkage and selection operator (LASSO) algorithm. Five machine learning (ML) models were trained to differentiate ccRCC from no-ccRCC, rad+cli nomogram was constructed based on clinical factors and radscore (radiomics score), and the performance of the classifier was mainly measured by area under the curve (AUC), accuracy, sensitivity, specificity, and F1. Finally, the ROC curves and radar plots were plotted according to the five performance parameters.

**Results:**

1130 radiomics features were extracted, there were 736 radiomics features with statistical differences were obtained, and 4 features were finally selected after the LASSO algorithm. In the validation set of this study, three of the five ML models (logistic regression, random forest and support vector machine) had excellent performance (AUC 0.9-1.0) and two models (adaptive boosting and decision tree) had good performance (AUC 0.7-0.9), all with accuracy ≥ 0.800. The rad+cli nomogram performance was found excellent in both the training set (AUC = 0.982,0.963-1.000, accuracy=0.941) and the validation set (AUC = 0.949,0.885-1.000, accuracy=0.911). The random forest model with perfect performance (AUC = 1, accuracy=1) was found superior compared to the model performance in the training set. The rad+cli nomogram model prevailed in the comparison of the model's performance in the validation set.

**Conclusion:**

The ML models and nomogram can be used to identify the relatively common pathological subtypes in clinic and provide some reference for clinicians.

## 1 INTRODUCTION

In 2012, with the surge of the third wave of artificial intelligence, Dutch scholar Lambin *et al*. [1] proposed the concept of radiomics, which can be used for tumor staging, identification, prognosis, gene mutation or receptor expression, *etc*. The process of radiomics [2] includes medical image acquisition, segmentation, extraction of features, model building and evaluation, *etc*. Many previous texture analysis platforms, such as Mazda, IBEX, ITK-SNAP, CEER, Artificial intelligent kit and Omni-Kinetics, *etc*., lack of standards in terms of repeatability of texture data. Some studies demonstrate the poor reproducibility of texture features under different texture platforms [3, 4]. In 2016, Zwanenburg *et al*. [5] published the requirements of the Image Biomarker Standardization Initiative. In 2017, Lambin *et al*. [6] suggested the need for standards in radiomics. In the same year, the Radiomics open-source website [7] was established to unify standards and specify guidelines for the standardization of various texture parameters, which were in accordance with the requirements of the Image Biomarker Standardization Initiative published by Zwanenburg *et al*. [5] in 2016. The radiomics official website supports only two texture feature extraction tools, including the 3Dslicer software radiomics plugin and the python language based pyradiomics package.

Renal cell carcinoma (RCC) is the most common solid lesion in the kidney, accounting for approximately 90% [8, 9] of all renal malignancies, the male-to-female ratio is 1.5:1 and the highest incidence is at the age of 60-70 years [10]. There are three main RCC types: ccRCC (70–80%), papillary renal cell carcinoma (pRCC) (types I and II, 10-15%, of which 60–70% are type I), and chromophobe RCC (chRCC) (4–5%) [9].⁠ The pRCC and chRCC subtypes exhibit comparatively better survival rates in comparison to ccRCC [11], there is no significant difference seen in outcome between patients with pRCC and chRCC types [11]. Thus, the categorization of RCC pathological subtypes is essential for clinical treatment.

Kocak *et al*. [12] used texture analysis of enhanced CT to tri-classify RCC subtypes, with an accuracy of 84.6% in the validation set when using an adaptive boosting artificial neural network algorithm. Han *et al*. [13] also used artificial neural network algorithms to identify three RCC subtypes in enhanced CT images and were able to achieve an AUC of 0.90. Li *et al*. [14] selected eight most relevant corticomedullary phase (CMP) texture features to build the machine learning (ML) model for differentiating ccRCC and non-ccRCC, which had an accuracy of 92.5% and an AUC of 0.95, moreover, they investigated the association between imaging features and VHL gene mutations. Wu *et al*. [15] used the well-known nnU-NET segmentation algorithm, achieved an AUC of 0.83 ± 0.1 for ccRCC and non-ccRCC classification efficacy, and an AUC of 0.80 ± 0.1 and 0.77 ± 0.1 for tumor grading and staging, respectively.

In this study, we identified the three most common types of pathology in clinical work, other classifications were not included in the study due to a wide variety and very small percentage or lack of specificity. Two additional pathological types (pRCC+chRCC) other than ccRCC were categorized as non-ccRCC, we explored the feasibility of a radiomics-based approach to differentiate ccRCC from non-ccRCC in order to provide a reference for the most common pathological classification decisions in clinical work.

## 2 PATIENTS AND METHODS

### 2.1 Patients

**Fig. (1):**
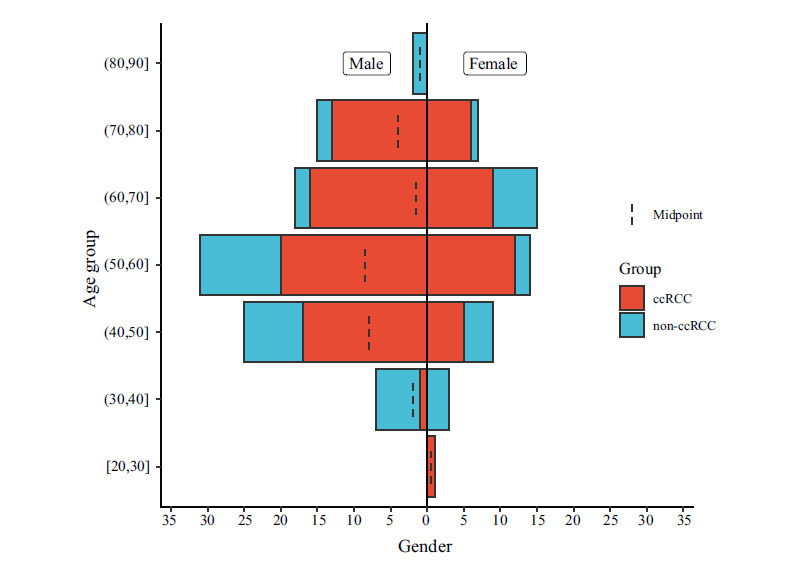
Age and sex distribution.

The ethical board approved the retrospective analysis. 147 patients with RCC underwent enhanced CT examinations of CMP and were confirmed by postoperative pathology from January 2012 to January 2021. Among them were 100 cases of ccRCC (67 males and 33 females, mean age, 58.56 ± 11.42 years, range, 29~80 years) and 47 cases of non-ccRCC (22chRCC+25pRCC, 31 males and 16 females; mean age, 53.06 ± 13.37 years, range, 31~82 years). Age was significantly different between the two groups (*P*=0.011) (Fig. **1**).

#### 2.1.1 Inclusion Criteria

(1) All patients who have underwent an enhanced CT scan in our hospital, (2) Patients who have not received surgery and chemoradiotherapy before CT scan, (3) Patients whose clinical, pathological, and imaging data were complete.

#### 2.1.2 Exclusion Criteria

(1) Those with poor image quality due to noise, artifacts, *etc*., (2) Patients whose pathological diagnosis was not renal cell carcinoma, (3) Patients with renal cell carcinoma containing two or more pathological types in one lesion.

### 2.2 Image Acquisition, Segmentation and Features Extraction

CT scanners from various manufacturers (Somatom Definition flash, Siemens, Germany, and Aquilion One, Aquilion PRIME, Toshiba, Japan) were used. The patient was scanned in a supine position with breath held from the top of the diaphragm to the level of the lower pole of both kidneys, and the enhanced scan was performed by injecting iophorol through the elbow vein with a high-pressure syringe at a flow rate of about 2.5~3.5 ml/s. The CMP images were obtained with a delay of 25~30 s after the injection.

The CT images were imported into the 3D-Slicer application from the hospital PACS in Dicom format. Two experienced diagnostic radiologists selected the region of interest (ROI) by examining each level of the kidney tumor. The ROI was first outlined layer by layer by the junior physician and reconstructed into the volume of interest (VOI), and finally reviewed by the senior physician, when there was a different view, the tumor boundary was discussed by other senior radiologists. After splitting the VOI, the features were extracted from the radiomics plugin of the 3D-slicer supported by Radiomics official website, the extraction method strictly followed the extraction rules provided by the Radiomics official website. These feature parameters complied with the requirements of the Image Biomarker Standardization Initiative published by Zwanenburg *et al*. [5] in 2016. All 8 kinds of radiomics features (Firstoder, GLCM, GLDM, GLRLM, GLSZM, NGTDM, shape3D, shape2D) [7] were extracted in the radiomics plugin of 3D-Slicer, and the total number of extracted features included the features of the original image and the features obtained after applying Gaussian Laplacian filter (LOG) and wavelet transformation. The final original Dicom file and VOI file were saved in NRRD format, and the extracted feature parameters were saved as an excel sheet in CSV format.

### 2.3 Statistical Analysis, Feature Selection, Prediction Model Building and Evaluation, Radscore Calculation and Nomogram Construction

#### 2.3.1 Feature Selection

R language version 4.1 was utilized for statistical analysis of the data. Foremost, the data were randomly grouped into training and validation sets according to 7:3, and then the training set was normalized, the normalization rule for the training set was extracted, and finally, the rule was applied to the validation set. Tests of homogeneity of variance (Levene test) and normality (Shapiro-Wilk test) were performed on the training set data. T-test was used for those that met normality and variance homogeneity, and T' test was used for those that met normality but not variance homogeneity. The Wilcoxon rank-sum test was used for any parameters that did not conform to normality. Parameters with statistical differences were preserved. Last, the least absolute shrinkage and selection operator (LASSO) regression was executed to choose the optimal features.

#### 2.3.2 Model Building and Evaluation

The optimal features from training set data were used to construct diagnostic models, and then assessed in the validation set data. The model construction and evaluation were performed with the R toolkits “pROC, Rcpp, rpart, rpart.plot, randomForest, e1071, adabag”, *etc*. In this study, five machine learning (ML) models, including logistic regression (LR), decision tree (DT), random forest (RF), support vector machine (SVM) and adaptive boosting (Adaboost), were used to identify the two different pathological sorts of tumors, and then their performance was assessed based on accuracy, sensitivity, specificity, F1 and AUC.

#### 2.3.3 Radscore Calculation

The radscore is a linear predictive value built by logistic regression, weighted according to each variable with corresponding weight coefficients. The final optimal features from training data were incorporated into the equation. The radscore was estimated for each patient in the training and validation sets through this formula.

#### 2.3.4 Nomogram Construction

Firstly, T, T', Chi-square, Fisher and Wilcoxon rank-sum tests were performed for clinical characteristics parameters. The clinical factors of the training set that were statistically different were included in logistic regression, which optimized applying the stepwise method, the method was applied to select the optimal parameters by removing some insignificant and collinear parameters. Next, the new version nomogram with the R language “regplot” package was plotted, which supported the glm function. Finally, ROC curves and radar plots were plotted to evaluate the effectiveness of the nomogram and the five ML models.

## 3 RESULTS

### 3.1 Feature Selection for Radiomics

**Fig. (2):**
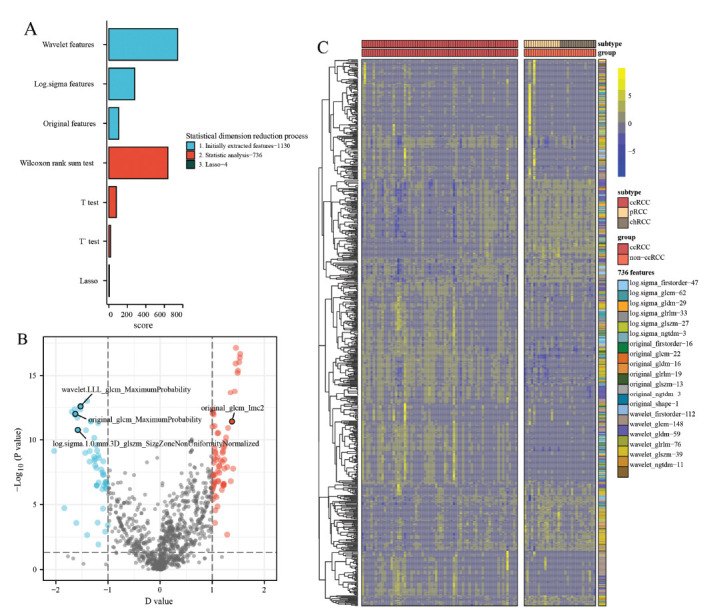
The process of feature dimensionality reduction. **(A)** The number of features corresponding to the feature downscaling process. **(B)** The vertical coordinate indicates the negative Log value of the *P* value, the smaller the *P* value corresponding to the negative Log value, the better the differentiation effectiveness. The horizontal coordinate represents the difference between the median (skewed distribution) or mean (normal distribution) of the two data characteristics of ccRCC and non-ccRCC, the value closer to 0 means that the data are more similar and the worse is the classification effect. **(C)** Heat map of 736 features after statistical analysis.

**Fig. (3):**
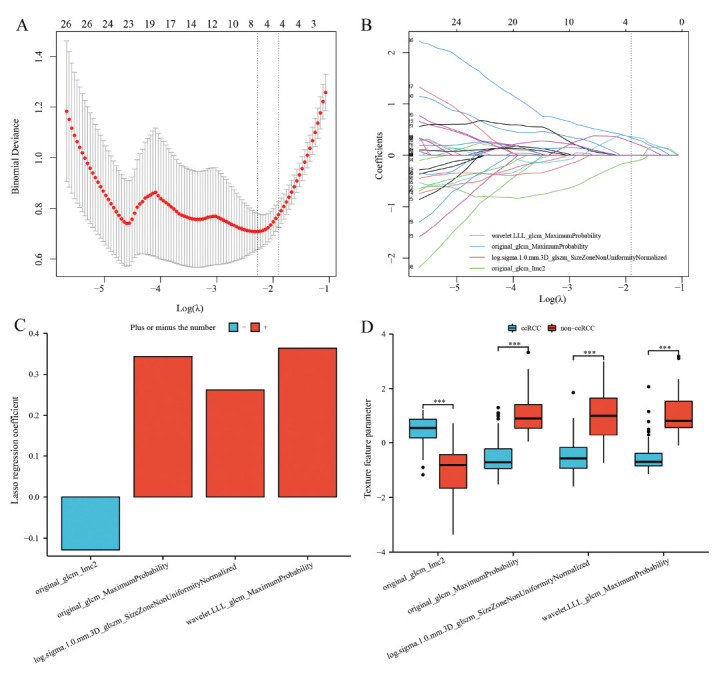
Feature selection using LASSO regression. **(A)** The 10-fold cross-validation (CV) process was repeated 100 times to generate the optimal penalization coefficient lambda (λ) in the LASSO model. Two vertical lines were traced at two log(λ) values, left one is the log(λ) value at the minimum binomial deviation (min criteria) and right one is the log(λ) value at one standard error away from the minimum binomial deviation (1-se criteria). 8 features and 4 features were selected, respectively, and λ was taken as 0.1491219 with log (λ)= -1.9029911867 (1-se criteria). **(B)** As the horizontal coordinate parameter log (λ) increased, the vertical coordinate parameter (coefficient) was found closer to zero. **(C)** LASSO regression coefficients for the 4 characteristics. (**D**) Box plots of the four features selected from the training set after standardization.

Fig. (**2A**) briefly describes the process of feature screening. 1168 features were collected and finally summarized in a CSV format table after discarding a portion of the top duplicate feature parameters that indicate version and configuration features, the last remaining features included 1130 features (107 original image features, 279 LOG pre-processed features, and 744 wavelet transformed features) (Table **1**). After data grouping and normalization, there were 736 statistically different features in the training set data (80 T-test, 18 T'-test, 638 Wilcoxon rank-sum test). The 736 features included 90 original features, 201 log.sigma features, and 445 wavelet features, the volcano diagram was made according to the -Log_10_P_value and D_value of the median (skewed distribution) or mean (normal distribution) (Fig. **2B**), also, the heat map of 736 features was made (Fig. **2C**). Thereafter, LASSO regression was used to decrease the dimensionality to pick the optimal features (Fig. **2A** and **3**), in Fig. (**3A**), the two dashed lines in the figure correspond to two criteria (min-criteria and 1-se criteria), corresponding to feature numbers of 8 and 4, respectively. 4 features were selected to prevent overfitting due to too many features. The corresponding λ was 0.1491219 and log (λ) was -1.9029911867 (Fig. **3A** and **B**). The features included original_glcm_Imc2, original_glcm_MaximumProbability, log.sigma.1.0.mm.3D_glszm_SizeZoneNonUniformityNormalized, and wavelet.LLL_glcm_MaximumProbability (Fig. **3B**). The corresponding LASSO regression coefficients were -0.1288100, 0.3433598, 0.2618784, and 0.3637253 (Fig. **3C**), respectively, and the box plots for the four characteristics are shown in Fig. (**3D**).

### 3.2 5 ML Models Effectiveness

The four features selected by LASSO based on the training set data were incorporated into five ML models, and the models were evaluated using parameters, such as AUC, specificity, sensitivity, accuracy, and F1. The effectiveness was perfect with AUC equal to 1, excellent with an AUC greater than 0.9, good with AUC between 0.7 and 0.9, and poor with AUC between 0.5 and 0.7. The evaluation parameters of the five ML classifiers are shown in Fig. (**4**) and Table **2**. The AUC of the training set in the five models (AdaBoost, DT, LR, RF and SVM) was 0.878, 0.987, 0.965, 1.000, and 0.971, respectively, and the accuracy of the training set was 0.902, 0.951, 0.882, 1.000, and 0.941, respectively. The AUC of the validation set was found to be 0.733, 0.851, 0.929, 0.918, and 0.918, respectively, and the accuracy of the validation set was obtained as 0.800, 0.867, 0.844, 0.867, and 0.867, respectively. From the performance of the 5 models in the validation set, three of the five ML models (LR, RF, and SVM) had excellent performance (AUC ≥ 0.9), and two models (Adaboost, DT) had good performance (0.7 < AUC < 0.9), and the accuracy of all models was greater than 0.800. The detailed metrics of AUC, specificity, sensitivity, accuracy, and F1 values for the five model efficacies are shown in Table **2**.

### 3.3 Building Nomograms Based on Clinical Characteristics and Radscore

#### 3.3.1 Radscore Formula

**Fig. (5):**
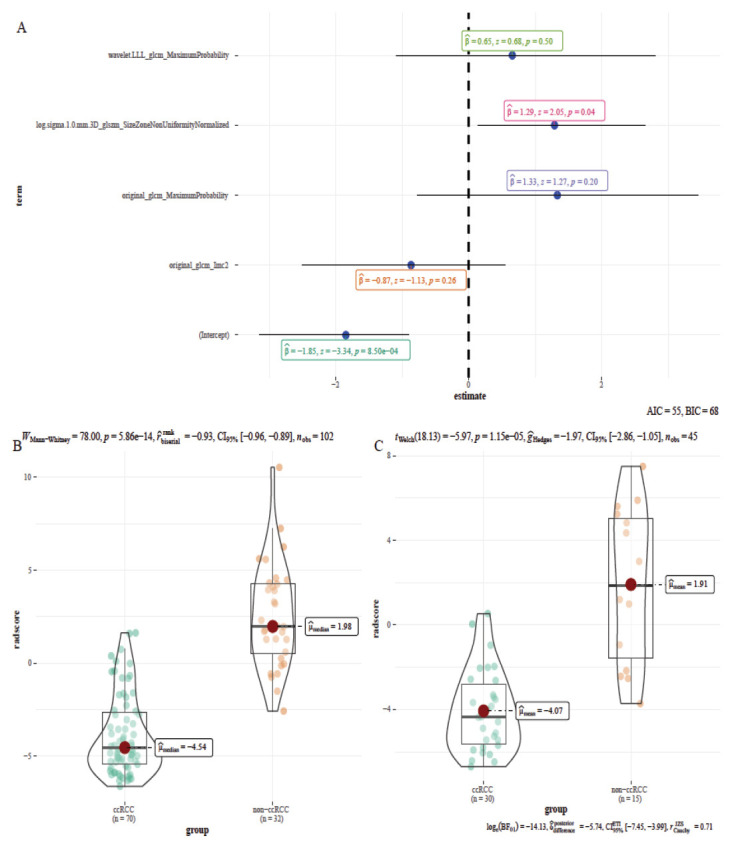
Radscore. **(A)** Graph of the weight coefficients of each variable of the radscore formula. **(B)** Radscore of training set. Mann-Whitney(U) test is also termed as the wilcoxon rank-sum test. **(C)** Radscore of validation set.

The four features selected by LASSO based on the training set data were included into the LR model, AIC was 55.106, and the radscore formula was constructed as follows (Fig. **5**):

Radscore = -1.8493 - 0.8679 * original_glcm_Imc2 + 1.3331* original_glcm_MaximumProbability + 1.2900 * log.sigma.1.0.mm.3D_glszm_SizeZoneNonUniformityNormalized + 0.6548 * wavelet.LLL_glcm_MaximumProbability.

The Radscore of the training set (Fig. **5B**) and validation set (Fig. **5C**) was calculated using this formula.

#### 3.3.2 Clinical Factors and Radscore

**Fig. (6):**
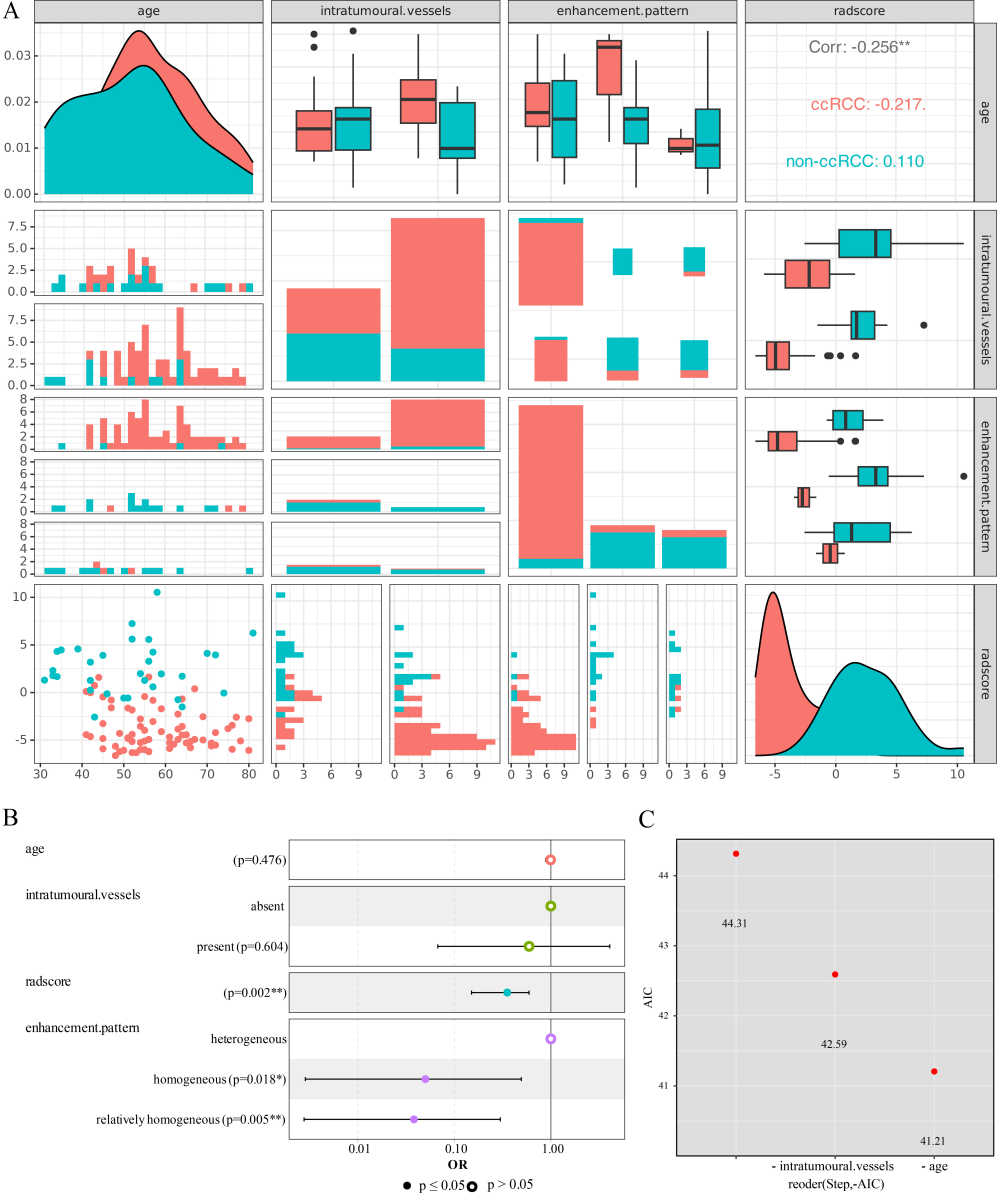
Training set clinical factor matrix diagram and stepwise regression screening for characteristics. **(A)** Training set clinical factor matrix diagram. **(B)** Forest map. **(C)** The variables of age and intratumoural vessels were eliminated using the stepwise method.

147 patients (100ccRCC+47non-ccRCC) were randomized 7:3 into a training set (n=102) and a validation set (n=45). The clinical factors of the training set showed statistically significant differences in four parameters (age, intratumoural vessels, enhancement pattern, and radscore) (Table **1**, Fig. **6A**). In Fig. (**6A**), it can be seen that age overlaps extensively in the two groups and is poorly classified, despite being statistically different. In addition, it can be seen in Fig. (**6A**) that the intratumoural vessels are significantly more predominant ccRCC than in blue non-ccRCC, the vast majority of red ccRCC showed a heterogeneous enhancement pattern, and the radscore of red ccRCC was mostly negative and the radscore of blue non-ccRCC was mostly positive (Fig. **6A**). Many scholars [16-18] have previously studied the efficacy of radscore for Fuhrman grading, and although our study did not reflect Fuhrman grading, the radscore in this study was statistically significantly different in terms of benign and malignant in ccRCC and non-ccRCC. It can be seen that the p-values of two parameters, age and intratumoural vessels, were greater than 0.05 in the case of the combination of 4 factors interacting with each other in the forest plot (Fig. **6B**). To remove the two variables, stepwise method was performed, and as the AIC decreased, age and intratumoural vessels were knocked out (Fig. **6B** and Fig. **6C**).

#### 3.3.3 Rad+cli Nomogram Construction and Evaluation

**Fig. (7):**
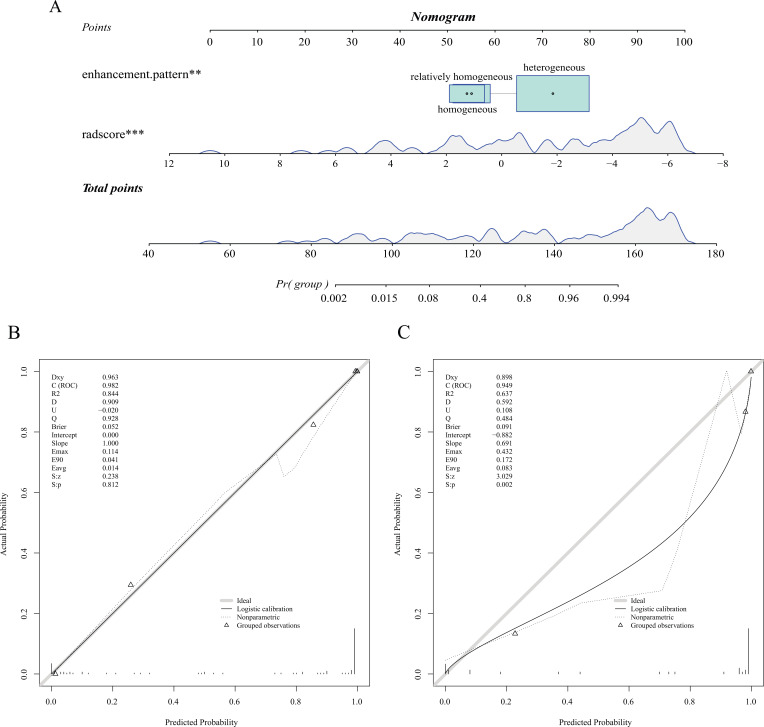
Rad+cli nomogram and calibration curve based on enhancement pattern and radscore after stepwise method to optimize variables. **(A)** Nomogram. **(B)** Training set calibration curve. **(C)** Validation set calibration curve.

The radscore was included in rad + cli (radscore and clinical factors) nomogram construction as a new variable with other clinical factors. ① After screening the variables using the stepwise method, enhancement pattern and radscore were incorporated in the logistic regression to build the rad+cli nomogram, then the calibration curves and ROC curves were plotted to visualize and assess the nomogram model. The C-index of training and validation sets was 0.982 and 0.949, respectively (Fig. **7**). The model effectiveness of the stepwise optimized nomogram was excellent in both the training set (AUC = 0.982,0.963-1.000, accuracy = 0.941) and the validation set (AUC = 0.949,0.885-1.000, accuracy = 0.911). ② When the effectiveness of five other ML models was compared, the RF model (AUC = 1, accuracy = 1) was found to be the most effective model in the training set, and the rad+cli nomogram model (AUC = 0.949, accuracy = 0.911) was found to be the best in the validation set. The other efficacy parameters are compared in Table **2** and Fig. (**8**).

## 4 DISCUSSION

This study incorporates a variety of mainstream ML models (AdaBoost, DT, LR, RF, SVM) with a new version nomogram. The new version nomogram can be established with glm function to avoid the disadvantages of the previous abbreviated version of nomogram, which can only be established with rms package and lrm function, many important parameters (C-index/AUC) may lead to different results due to the different algorithms between lrm and glm function. This was an improvement over previous articles on nomogram in terms of the reproducibility of data. When pre-processing the data, we first grouped the data, and then normalized the training set and applied the normalization rules to the validation set, if there is a new batch of data, after normalization according to the rule of the training set, the new data features can be correctly fed back.

The RF model has shown perfect performance in the training set (AUC=1, accuracy=1) and excellent performance in the validation set, mainly because the algorithm principle itself has two stochastic processes, therefore, it is not easy to produce overfitting. However, the performance of different DT models in the model varies somewhat, but all DT models have the same voting weight, and the overall prediction stability of RF is affected by it. There have been studies conducted to improve the process of RF, such as the weighted random forest algorithm [19] proposed by weighting the decision tree hierarchy according to the decision tree efficacy, weighted methods for voting improvement [20], weighted methods for random selection of features [21], *etc*.

Generally when using LASSO to filter features, the number of features corresponding to the min criterion is greater than the number of features of the 1-se criterion. In this study, we tested the whole process a dozen times using different random grouping seeds, we found that when the number of features finally selected was relatively small, the features obtained by direct LASSO remained the same as those obtained after statistical analysis and then using LASSO, with only minor differences in the regression coefficients. The vast majority of the cases were the same, and only a few appeared to be different. If there was a statistical error, the two results may not be duplicated. We can draw a possible conclusion that LASSO can be performed directly on the pre-processed data, because the LASSO algorithm can compress the weighting coefficients of insignificant variables to 0 (Fig. **3B**), it is suitable for dimensionality reduction of high-dimensional data. Previously, some scholars have directly used LASSO dimensionality reduction [22, 23].

Among the clinical factors of training set in our study, age, intratumoural vessels, enhancement pattern and radscore were statistically different, age had the largest p-value and the worst classification effect, which can be seen by the large overlap of age in the two groups (Fig. **6A**, Table **1**), and the p-values of age and intratumoural vessels became larger than 0.2 in the forest plot where the four variables interacted with each other (Fig. **6B**). Brian Meehan *et al*. [24] found that the vascular characteristics of renal tumors exhibited possible age-related alterations, with higher microvessel density and endothelial nitric oxide synthase (eNOS) positivity in patients over 65 years than in younger patients under 65 years, and vascular DLL1 expression was more prevalent. A previous study involving 490 patients [25] with renal cell carcinoma found higher age to be associated with higher Fhrman nuclear grading (*P*<0.001), and histological type was associated with age (*P*=0.016). There is plentiful neovascularization in ccRCC [26], and tumor enhancement correlates with microvessel density [27] and Fuhrman grading, and the microvessel area of immature vessels is positively associated with RCC tumor grade, stage, metastasis and prognosis [28]. This may make ccRCC to exhibit more obvious enhancement in CMP, with image brightness cross-draft and texture distribution more disorderly. The two factors, intratumoural vessels and enhancement pattern, may have some co-linearity and correlation, and exhibit variable redundancy, finally, age and intratumoural vessels were filtered out as a confounding factor during the stepwise screening of variables by logistic regression.

In this study, ccRCC and non-ccRCC were distinguished from each other using a texture analysis method. RCC was also more difficult to identify with fat-poor angiomyolipoma (fp-AML) and renal oncocytoma (RO). The proportion of benign kidney tumors was relatively low, among them, angiomyolipoma (AML) and RO were the most common benign tumors, and 5% of AML were fat deficient termed as fp-AML, with no obvious necrotic cystic hemorrhage similar to the homogeneous ccRCC presentation. RO accounted for 3-7% [29] of all renal tumors, of these, 46% had a central scar and 26% of ccRCC also presented with such a scar [30]. Also, the chRCC and RO have many commonalities in morphologic, histologic, electron microscopic and immunohistochemical features, with many overlapping imaging features.

This study has some limitations. At first, we have identified the three most common pathological types of RCC in current usual clinical work, the other classifications were not included in the study due to the lack of specificity of each small percentage of more classifications, and there may be some bias. Second, all pathological types were confirmed by postoperative pathology, but the accuracy of pathological diagnosis was not exactly 100%, there were also difficulties in differentiating between pathological types of renal cell carcinoma and other tumors on electron or light microscopic pathological images, and the accuracy was not 100% after combining with immunohistochemistry. Third, the pyradiomics forum is significantly more popular than the 3D-slicer radiomics forum, many of the latest updates are not implemented in 3D-slicer's radiomics plugin. Lastly, the details of radiomics research are troublesome, there is no automated process, which is an obstacle for radiology to reach practical application, and also, there are many gaps and deficiencies in our study that will be added and corrected in future studies.

## CONCLUSION

The ML models and nomogram can be used to identify the relatively common pathological subtypes in clinic and provide some reference for clinicians.

## Figures and Tables

**Fig. (4):**
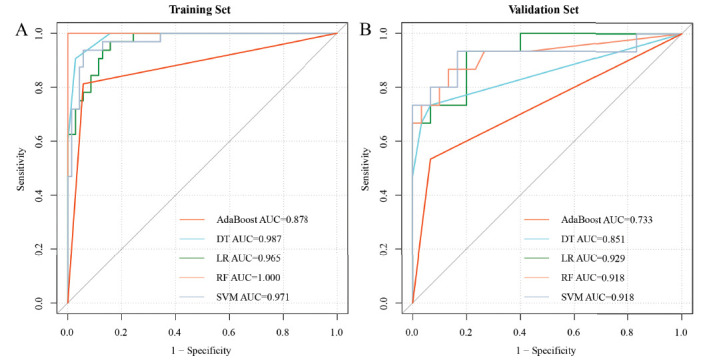
Comparison of AUC for five ML models in training (**A**) and validation sets (**B**). In the validation set, LR, RF, and SVM models were found to have excellent performance (AUC0.9-1.0), while AdaBoost and DT models with good performance (AUC0.7-0.9).

**Fig. (8):**
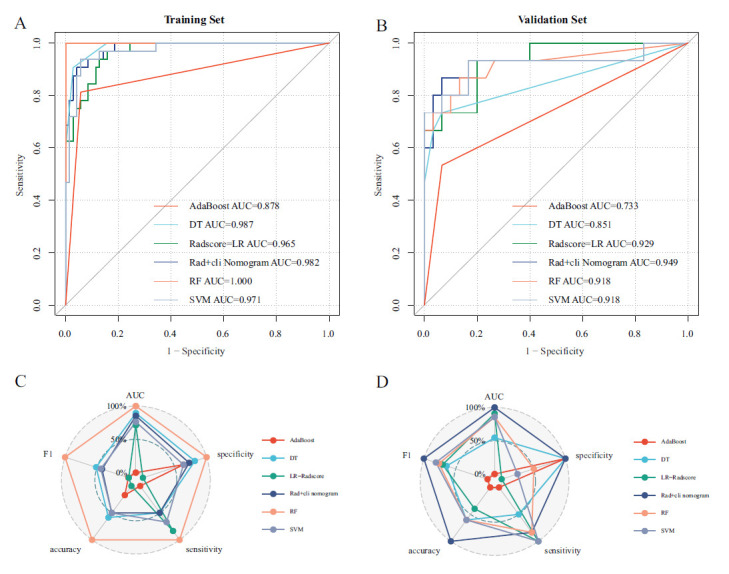
ROC curve and normalized radar plot for nomogram and five ML models in training and validation sets. The nomogram and five ML models' efficiency parameters were mainly concentrated at 0.8-1.0 in the training set and at 0.5-1.0 in the validation set. The radar plots of the original values may be particularly tight at certain intervals resulting in poor differentiation, especially in the training set, making the radar plot differentiation obvious, the normalized radar plot reassigned the parameters in the interval 0-1 according to the maximum and minimum values. (**A**) The ROC of the nomogram and 5 ML models in the training set. (**B**) The ROC of the nomogram and 5 ML models in the validation set. (**C**) The RF model exhibited the best efficacy in the training set. (**D**) The Rad+cli nomogram showed the best efficacy in the validation set.

**Table 1:** Clinical factors of the training and validation sets.

**Characteristic**	**Training Set (n=102)**		**-**	**Validation Set (n=45)**		**-**
	**ccRCC**	**Non-ccRCC**	***P***	**ccRCC**	**Non-ccRCC**	***P***
	**(n=70)**	**(n=32)**	**-**	**(n=30)**	**(n=15)**	**-**
**Age, mean ± SD**	58.07 ± 10.46	51.38 ± 13.16	0.007	59.7 ± 13.53	56.67 ± 13.55	0.482
**Gender, n (%)**	-	-	0.401	-	-	0.283
Female	23 (22.5%)	14 (13.7%)	-	10 (22.2%)	2 (4.4%)	-
Male	47 (46.1%)	18 (17.6%)	-	20 (44.4%)	13 (28.9%)	-
**Intratumoural vessels, n (%)**	-	-	0.002	-	-	<0.001
Absent	18 (17.6%)	19 (18.6%)	-	7 (15.6%)	12 (26.7%)	-
Present	52 (51%)	13 (12.7%)		23 (51.1%)	3 (6.7%)	-
**Enhancement pattern, n (%)**	-	-	< 0.001	-	-	< 0.001
Heterogeneous	64 (62.7%)	4 (3.9%)		29 (64.4%)	4 (8.9%)	-
Homogeneous	3 (2.9%)	15 (14.7%)	-	0 (0%)	6 (13.3%)	-
Relatively homogeneous	3 (2.9%)	13 (12.7%)	-	1 (2.2%)	5 (11.1%)	-
**Renal vein invasion, n (%)**	-	-	0.166	-	-	0.540
Absent	60 (58.8%)	31 (30.4%)	-	27 (60%)	15 (33.3%)	-
Present	10 (9.8%)	1 (1%)	-	3 (6.7%)	0 (0%)	-
Tumor size (cm2), median (IQR)	16.24 (7.28, 28.97)	11.22 (4.67, 21.28)	0.080	14.39 (8.12, 29.7)	16.28 (2.59, 28.6)	0.745
Radscore, median (IQR)	-4.54 (-5.4, -2.63)	1.98 (0.51, 4.26)	< 0.001	-4.07 ± 1.94	1.91 ± 3.62	< 0.001

**Table 2:** Effectiveness of 5 ML models and nomogram.

**Models**	**Group**	**Auc (95%CL)**	**Specificity**	**Sensitivity**	**Accuracy**	**F1**
AdaBoost	Train	0.878(0.804-0.952)	0.943	0.813	0.902	0.839
	Validation	0.733(0.595-0.872)	0.933	0.533	0.800	0.640
DT	Train	0.987(0.973-1.000)	0.971	0.906	0.951	0.921
	Validation	0.851(0.728-0.975)	0.933	0.733	0.867	0.786
LR = Radscore	Train	0.965(0.936-0.994)	0.843	0.969	0.882	0.838
	Validation	0.929(0.855-1.000)	0.800	0.933	0.844	0.800
RF	Train	1.000(1.000-1.000)	1.000	1.000	1.000	1.000
	Validation	0.918(0.817-1.000)	0.867	0.867	0.867	0.813
SVM	Train	0.971(0.943-1.000)	0.943	0.938	0.941	0.909
	Validation	0.918(0.806-1.000)	0.833	0.933	0.867	0.824
Rad+cli nomogram	Train	0.982(0.963-1.000)	0.957	0.906	0.941	0.906
	Validation	0.949(0.885-1.000)	0.933	0.867	0.911	0.867

## Data Availability

The datasets used and/or analyzed during the current study are not publicly available due to patient privacy but are available from the corresponding author on reasonable request.

## LIST OF ABBREVIATIONS

Adaboost: Adaptive Boosting
AML: Angiomyolipoma
AUC: Area Under the Curve
ccRCC: Clear Cell Renal Cell Carcinoma
chRCC: Chromophobe Renal Cell Carcinoma
CMP: Corticomedullary Phase
DT: Decision Tree
eNOS: Endothelial Nitric Oxide Synthase
fp-AML: Fat-Poor Angiomyolipoma
LASSO: Least Absolute Shrinkage and Selection Operator
LR: Logistic Regression
ML: Machine Learning
non-ccRCC: Non-Clear-Cell Renal Cell Carcinoma
pRCC: Papillary Renal Cell Carcinoma
RCC: Renal Cell Carcinoma
RO: Renal Oncocytoma
ROC: Receiver Operating Curve
ROI: Region of Interest
SVM: Support Vector Machine
VOI: Volumes of Interest
